# The prevalence and risk factors for pterygium in South Korea: the Korea National Health and Nutrition Examination Survey (KNHANES) 2009-2010

**DOI:** 10.4178/epih.e2016015

**Published:** 2016-04-29

**Authors:** Eun-Young Pyo, Gui Hyeong Mun, Kyung Chul Yoon

**Affiliations:** 1 Department of Nursing, Kyung-In Women’s University, Inchon, Korea; 2 Department of Ophthalmology, Chonnam National University Medical School, Gwangju, Korea

**Keywords:** National Health and Nutrition Examination Survey, Prevalence, Pterygium, Risk factors, Republic of Korea

## Abstract

**OBJECTIVES:**

To determine the prevalence and risk factors for pterygium in the adult Korean population of South Korea.

**METHODS:**

Data were analyzed from 9,193 participants who were 40 years of age or older from the Korea National Health and Nutrition Examination Survey (KNHANES), conducted from 2009 to 2010. Standardized slit-lamp examinations were performed by study ophthalmologists to examine the anterior segment for evidence of pterygium. Pterygium was graded clinically as T1 (atrophic), T2 (intermediate), or T3 (opaque). Every participant underwent ocular and systemic examinations, as well as interviewer-administered questionnaires. Any evidence of pterygium and observed association between the risk factors were recorded.

**RESULTS:**

The mean age of the subjects was 55.7 (±0.2) years. Of the 9,193 eligible subjects, 935 had pterygium in at least one eye. In a multivariate logistic regression analysis adjusted for age and sex, pterygium was significantly associated with rural vs. urban residence (odds ratio [OR], 1.7; 95% confidence interval [CI], 1.4 to 2.0), lower level of education (OR, 4.5; 95% CI, 3.1 to 6.6), low income (OR, 1.3; 95% CI, 1.0 to 1.8), smoking (OR, 0.7; 95% CI, 0.5 to 1.0), and more hours of sun exposure (OR, 1.5; 95% CI, 1.2 to 1.8). After adjusting for all variables, the prevalence of pterygium was significantly associated with age, sex, residence, education level, and smoking.

**CONCLUSIONS:**

This is a nationwide epidemiologic study in South Korea to assess the prevalence of and risk factors for pterygium. The overall prevalence of pterygium was 8.8% among Koreans aged 40 years or older. Older age, male gender, rural residence, lower level of education, and non-smoking were associated with the development of pterygium.

## INTRODUCTION

It is well known that pterygium is one of the most common eye diseases. It is an abnormal, proliferative overgrowth of fibrovascular tissue which develops from the bulbar conjunctiva in the cornea. In advanced cases, it can induce significant astigmatism and decrease visual function caused by loss of corneal transparency [1,2]. According to previous studies, residence, age, race, sex, sunlight exposure, and education level were associated with risk for pterygium [3-18]. At the moment the exact the pathogenesis of pterygium is not clear.

The prevalence of pterygium has been reported in several population-based studies [7-20]. The prevalence rates of pterygium mentioned in different studies are greatly different from 2.8% to 33.0%. There have been several epidemiological studies conducted in South Korea (hereafter Korea) for several age-related eye diseases [17,18,21-23]. But a population-based research on large scale of the rate of pterygium and other eye diseases has not been studied until now. A systematic health survey in Korea, the Korea National Health and Nutrition Examination Survey (KNHANES) was started in 1998. Studies analyzing the KNHANES data have shown that the prevalence of pterygium was 6.2% to 8.9% [17,18].

This study aims to examine the national prevalence of pterygium in an adult Korean population of Korea, based on survey data which is obtained from the KNHANES, and to try to analyze the risk factor which is associated with the occurrence of pterygium.

## MATERIALS AND METHODS

### Study design and population

The study of the prevalence of pterygium is based on a population-based survey on eye diseases by the KNHANES during 2009 to 2010. The KNHANES is a population-based, cross-sectional epidemiological survey of Korea which has been performed since 1998. 4,000 households in 200 enumeration districts were annually selected by the panel to represent the general Korean population by using a multistage clustered and stratified random sampling method that was based on the data from the 2005 National Census [19]. Participating household members were interviewed state of health and nutrition and were supposed to have a basic health examination such as blood pressure, blood and urine tests, ear and nose examination, a pulmonary function test, and a dental examination. When the Korean Ophthalmologic Society began to participate in this project beginning in 2008, ophthalmologic interviews and examinations were also performed on the same participants [19]. The participants of this survey were adults aged 40 years or older in Korea. From this sampling method, the initial sample size was 9,365 participants. Eventually, 9,193 individuals (response rate 98.2%) were surveyed and comprehensive ocular examinations were conducted. This study tried to analyze publicly available data sets, which allows it to be exempted from institutional review board approval.

### Examination methods and the definition of pterygium

Each participant had a comprehensive vision and eye examination, which included: presenting and best-corrected visual acuity, auto-refraction, and slit-lamp examination. A structured slit-lamp examination (BQ-900, Haag-Streit AG, Koeniz, Switzerland) was done by trained ophthalmologists in order to examine the anterior segment for evidence of pterygium. The diagnosis of pterygium was made clinically, and it was defined as a radically oriented fibrovascular lesion crossing over the nasal or temporal limbus. Grading was based on the visibility of the underlying episcleral blood vessels [25]. T1 (atrophic) is defined as episcleral vessels clearly visible, T2 (intermediate) as vessels partially visible, and T3 (opaque) as vessels wholly obscured. They were also classified as either unilateral or bilateral, and the position of the pterygium in either eye was recorded. The size of the pterygium was measured as the greatest distance from the limbus to the apex of the lesion. A subject was defined as positive for pterygium if at least one pterygium lesion was confirmed in either eye.

### Risk factor assessment

A detailed interviewer-administered questionnaire was deployed to collect relevant sociodemographic and medical information. Collected data included the information such as marital status, education, occupation, current housing status, lifestyle, eye symptoms, past medical and surgical history, and family history of eye disease. Education level was categorized into four groups: elementary school or less, middle school, high school, and college/university or more. The revised household income was calculated from the total household income divided by family size. A participant was considered to be a ‘drinker’ if he or she drank more than once per month over the past year. Smoking status was divided into two groups: smokers and non-smokers. Non-smokers indicate people who have never smoked or have smoked less than five packs in their lifetime. Height and weight were obtained using standardized techniques and equipment. Body mass index (BMI) was calculated as the ratio of weight to the square of height (kg/m^2^). Systolic blood pressure (SBP) and diastolic blood pressure (DBP) were measured by standard methods using a sphygmomanometer with the patient in a sitting position. The SBP and DBP readings were twice recorded at five minutes intervals and were averaged for in this analysis. Blood samples were obtained after fasting for 12 hours in the morning. Biochemical markers like fasting plasma glucose, total cholesterol, triglycerides, and high-density lipoprotein cholesterol were measured. Diabetes mellitus (DM) was defined as a fasting plasma glucose level above 126 mg/dL, and/ or treatment with oral hypoglycemic agents or insulin. Hypertension was defined as an SBP over 140 mmHg, and/or DBP over 90 mmHg, or by a self-reported current use of antihypertensive medications. Hypercholesterolemia was defined as total cholesterol over 200 mg/dL.

### Statistical analysis

Continuous variables were described as mean±standard error (SE), while discrete variables were expressed as numbers and percentages. Differences in categorical data were analyzed by using the chi-square test. The relevance of all variables and the risk factors of pterygium were estimated by the odds ratio (OR) and its 95% confidence intervals (CI). Multiple logistic regression models were used to evaluate the independent effects of the variables on the risk factors for pterygium. Statistical analyses were performed with complex-samples analysis procedures in SPSS version 18.0 (SPSS Inc., Chicago, IL, USA). We used the KNHANES stratification variables and sampling weights designated by the Korea Centers for Disease Control and Prevention (KCDC). All data of p<0.05 was considered statistically significant.

## RESULTS

The social and demographic characteristics of the participants are summarized in Table 1. The total 9,193 people among 9,365 eligible subjects who are aged 40 years and older participated in this survey. Of these 9,193 participants, 4,003 were male while 5,190 were female. The participants mean age was 55.7±0.2 years (54.7±0.3 years for male; 56.6±0.3 years for female). The overall prevalence of pterygium in either eye was 8.8%, and the rate of bilateral pterygium was 2.7%.

There were 935 subjects with pterygium in either eye; 467 subjects were male and 468 subjects were female. A significant increasing trend was found in the prevalence of pterygium with age (p<0.001), which was consistently higher in male compared with female subjects (*p*=0.003) (Table 2).

Of the 935 subjects having pterygium in either eye, 449 subjects were categorized as having grade T1, 361 subjects had grade T2, and the rest 123 subjects had grade T3 (Table 3). An increase in prevalence depending on old age was observed for subjects with grade T1 and T2 pterygium (p<0.001, *p*=0.01, respectively). In contrast, a significant increasing trend was not found in the prevalence of grade T3 pterygium with increasing age. In all three groups, significant sex difference was not found according to the grade of pterygium.

The results of univariate and multivariate logistic regression analysis are presented in Table 4. Univariate logistic regression analysis revealed that the presence of pterygium was associated with older subjects, males, rural vs. urban residence, lower level of education, low income, more hours of exposure to sunlight, and hypertension. After adjusting for age and sex, the prevalence of pterygium was significantly associated with rural vs. urban residence (OR, 1.7; 95% CI, 1.4 to 2.0), lower level of education (OR, 4.5; 95% CI, 3.1 to 6.6 for elementary school or less vs. college/university or more), low income (OR, 1.3; 95% CI, 1.0 to 1.8 for first quartile vs. fourth quartile), smoking (OR, 0.7; 95% CI, 0.5 to 1.0), and more hours of exposure to sunlight (OR, 1.5; 95% CI, 1.2 to 1.8). After adjusting for all variables, the prevalence of pterygium was found to be significantly associated with age, sex, residence, level of education, and smoking (Table 4). Interestingly, after adjusting for all variables using a multiple regression analysis, income level and sun exposure are not risk factors for pterygium.

## DISCUSSION

The prevalence rates of pterygium reported in different studies vary greatly from 2.8% to 33.0% [7-16,19,20]. 8.8% of Koreans adult who are over 40 years were found to have pterygium in either eye, with 2.7% having pterygium in both eyes. The highest prevalence of pterygium in a Chinese people who are over 50 years in Doumen county, Southern China was 33.0% [11]. In Japanese population who are over 40 years in Kumejima, it was 30.8% [13]. On the other hand, according to three population-based studies of Malays, Indians, and Chinese people who are over 40 years, the prevalence of pterygium in Singapore was 10.1% [20], which was similar to the results of our survey. The lowest prevalence of pterygium was 2.83%, reported in an Australian population in Victoria, who are over 40 years [7], and 2.9% in the Chinese people in Beijing aged 40 years and older [15]. Rim et al. [17] and Lim et al. [18] performed studies on the basis of the KNHANES that showed that the prevalence of pterygium was 6.2% to 6.7%, but this current study had different results. This divergent result was caused by age difference and the difference in the affected parts of the eyes of each participant. In addition, the nationality of the participants also had an influence on the prevalence of pterygium.

The previous studies [26-28] showed that ultraviolet exposure is related to development of pterygium, which suggest that the prevalence of pterygium in a population is directly associated with the proximity of that population to the equator. They also found that there was so called a “pterygium belt” which is located between 37º north and south of the equator. However, this hypothesis may be overly simplistic. But it is hard to say that the prevalence of pterygium is not necessarily related to latitude. Similar prevalences of pterygium have been observed in Singapore (1º N) and Korea (37º N), which are located 36º apart in latitude [20]. In contrast, there was a difference in the prevalence of pterygium between Beijing (39º N) and Korea (37º N) despite their similar latitude [15]. Focusing solely on ultraviolet exposure to explain the variation in the prevalence of pterygium in different populations may brings us the difficulty in explaining the effects of other potential risks factors, such as possible genetic predisposition to the disease, lifestyle, and other ocular and systemic diseases.

This study also confirms that age was an important factor for the formation of pterygium, which is accords with the findings of many previous researches [6-15,17,18]. The association of sex with the development of pterygium is debatable. We found statistical significance of a difference in development of pterygium between male and female subjects. Female subjects were at lower risk than males, which was consistent with that of many previous studies [7,8,10,12-15,19]. In contrast, in the reports from the two studies done in China, female subjects were found to have a higher risk than males [11,20]. In Indonesia, there was no sex difference in the development of pterygium [9]. This result suggests there might be variations between different populations. Consequently we should take care of direct comparisons of prevalence rates of between studies.

Although it is difficult to quantify the true amount of one’s exposure to sunlight with ultraviolet radiation, many studies have shown that outdoor workers have a higher risk of the development of pterygium, with cumulative exposure to ultraviolet radiation [3-7,9,13,16-18]. In common with other studies, the development of pterygium in the Korean population increased with more hours of sun exposure [17,18]. In addition, development of pterygium in the Korean population was independently associated with the residence in a rural area, and the lower level of education, which implicates their life would correlate with outdoor work. Probably people living in rural regions and with lower levels of education are more likely to have an outdoor job. Interestingly, after adjusting for all variables using a multiple regression analysis, income level and sun exposure are not risk factors for pterygium.

In this research, smoking was protective against pterygium. Other studies also showed that smokers were less likely to have pterygium in other studies as well [7,9]. How smoking might be protective for pterygium remains unclear. However, the effect of smoking in inducing immune suppression is thought to explain the beneficial effect of smoking on some immunologically mediated disorders [29].

Several population-based studies demonstrated an association between the development of pterygium and higher blood pressure and total cholesterol level [14,30]. Peiretti et al. [30] have reported a high expression of low-density lipoprotein receptors with an altered cholesterol metabolism in pterygium tissues, consistent with other tumor-like tissues. Whereas, in this study, systemic disease, including hypertension, hyperlipidemia, and DM, did not show any association with the development of pterygium.

This study is the first epidemiologic study that has investigated the prevalence of pterygium in Korea based on the nationally representative data from a government-sponsored survey. The strengths of this survey is that it includes its nationwide, large sample size with standardized ocular assessment by ophthalmologists, using an objective method of grading pterygium and comprehensibly assessing associated factors. Our research revealed that significant racial/ethnic variations may exist even within a single Asian country. Our study may contribute to provide further insights into etiology, ethnic differences and the impact of pterygium on public health in Korean population. Despite of these strong points, our study has some limitation in explaining sunlight exposure models in detail and in cumulating occupational history. In the Barbados Eye Study, using sunglasses and prescription glasses can lower frequency of pterygium [16]. Presumably, the use of sunglasses or hat blocks can reduce ultraviolet radiation, helping decrease the development of pterygium. The frequency of use of sunglasses and hats may influence on these effects. In our study, investigation of ultraviolet radiation exposure was derived from a questionnaire rather than from objective measurement. Therefore, using a standardized questionnaire and objective measurement of sunlight exposure is needed in further studies.

Our attempt is the first nationwide epidemiologic study which has been conducted by both the Ophthalmologic Society and government organizations. This method proved powerful in investigating the national prevalence of disease conditions. Relation to pterygium with older age, males, and rural vs. urban residence, lower levels of education, and nonsmoking were also found. These results may allow ophthalmologists and nurses to counsel patients about potential modifiable risk factors.

## Figures and Tables

**Table 1.** The social and demographic features of participants

Characteristics	Total (n=9,193)	Any pterygium (n=935)	Bilateral pterygium (n=290)
Age (yr)			
40-49	2,639	95 (3.6)	27 (0.9)
50-59	2,403	189 (8.0)	57 (2.5)
60-69	2,216	293 (12.9)	82 (3.7)
≥70	1,935	358 (18.2)	124 (7.3)
p-value		<0.001	<0.001
Sex			
Male	4,003	467 (9.8)	147 (3.1)
Female	5,190	468 (7.9)	143 (2.5)
p-value		0.003	0.11
Residence			
Urban	6,564	526 (7.2)	162 (2.3)
Rural	2,629	409 (13.6)	128 (4.1)
p-value		<0.001	0.001
Education			
College/university or more	3,581	581 (15.1)	198 (5.7)
High school	1,407	148 (9.4)	40 (2.6)
Middle school	2,479	140 (5.6)	34 (1.2)
Elementary school or less	1,605	46 (2.7)	10 (0.6)
p-value		<0.001	< 0.001
Income (quartile)^1^			
1st	2,459	374 (13.6)	120 (4.5)
2nd	2,167	241 (9.4)	77 (3.2)
3rd	2,166	167 (7.0)	42 (1.5)
4th	2,279	142 (5.9)	47 (2.1)
p-value		<0.001	< 0.001
Alcohol consumption			
No	4,705	522 (9.5)	171 (3.3)
Yes	4,355	398 (8.2)	115 (2.3)
p-value		0.07	0.01
Smoking			
Non-smoker	5,471	531 (8.5)	162 (2.6)
Smoker	3,610	390 (9.2)	125 (3.0)
p-value		0.31	0.29
Sunlight exposure (hr)			
<5	6,696	547 (7.5)	168 (2.3)
≥5	2,476	387 (12.8)	122 (4.3)
p-value		<0.001	< 0.001
Obesity (body mass index, kg/m^2^)			
< 25	5,993	612 (8.6)	191 (2.9)
≥ 25	3,184	321 (9.0)	98 (2.6)
p-value		0.61	0.42
Diabetes mellitus			
No	7,241	709 (8.4)	220 (2.6)
Yes	1,154	134 (10.1)	45 (3.6)
p-value		0.13	0.10
Hypertension			
No	4,976	419 (7.0)	127 (2.0)
Yes	4,119	506 (11.2)	160 (3.8)
p-value		<0.001	< 0.001
Hypercholesterolemia			
No	7,053	719 (8.8)	35 (2.3)
Yes	1,362	131 (8.2)	232 (2.8)
p-value		0.52	0.31

Values are presented as number (%). p-values based on analysis of variance or chi-square test as appropriate.

1 Equivalised household income weighted by family number.

**Table 2.** The age- and sex-specific prevalence of pterygium

Classification	n	% (SE)	Age (yr)				p-value for trend for age	p-value for sex
			40-49	50-59	60-69	≥70		
Any pterygium								
All	935	8.8 (0.4)	3.6(0.5)	8.0 (0.8)	12.9 (0.8)	18.2 (1.1)	<0.001	0.003
Male	467	9.8 (0.6)	4.6 (0.8)	9.6(1.2)	15.3 (1.4)	18.7 (1.6)	<0.001	
Female	468	7.9 (0.5)	2.5 (0.5)	6.4 (0.9)	10.8 (1.0)	17.8 (1.4)	<0.001	

SE, standard error.

**Table 3.** The age- and sex-specific proportion rates according to the grades of pterygium

Pterygium	n	% (SE)	Age (yr)				p-value for trend for age	p-value for sex
			40-49	50-59	60-69	≥70		
T1								0.41
All	449	50.5 (2.5)	64.8 (5.7)	59.3 (3.9)	41.6 (3.9)	43.7 (3.3)	<0.001	
Male	219	49.1 (2.9)	64.6 (7.3)	54.4 (5.2)	37.8 (4.8)	43.2 (4.6)	0.006	
Female	230	52.2 (3.4)	65.0 (8.9)	66.5 (5.8)	46.5 (6.2)	44.1 (4.5)	0.007	
T2								0.41
All	361	37.5 (2.2)	26.7 (5.2)	32.1 (3.7)	44.4 (3.9)	41.4 (3.3)	0.01	
Male	186	39.0 (2.8)	27.2 (6.9)	34.6 (5.0)	49.5 (5.0)	41.6 (4.5)	0.04	
Female	175	35.8 (3.1)	25.6 (7.3)	28.5 (5.4)	37.8 (5.9)	41.3 (4.6)	0.17	
T3								0.96
All	123	12.0 (1.5)	8.6 (3.2)	8.6(2.7)	14.0 (2.5)	14.9 (2.5)	0.23	
Male	61	11.9 (1.9)	8.2 (4.0)	11.0 (4.1)	12.7 (3.1)	15.2 (3.6)	0.66	
Female	62	12.1 (1.5)	9.4 (5.5)	5.0 (2.8)	15.6 (4.1)	14.6 (3.4)	0.19	

SE, standard error; T1, atrophic: episcleral vessels clearly visible; T2, intermediate: vessels partially visible; T3, opaque: vessels wholly obscured.

**Table 4.** The risk factors associated with any pterygium and a multivariate analysis of factors associated with any pterygium

Characteristics	Crude	p-value	Age-sex adjusted	p-value	Adjusted	p-value
Age (yr)						
40-49	1.0 (reference)				1.0 (reference)	
50-59	2.3 (l.8, 3.1)	<0.001			1.6 (1.1, 2.1)	0.008
60-69	4.0 (3.0, 5.4)	<0.001			2.1 (1.5, 2.9)	< 0.001
≥70	6.0 (4.5, 8.1)	<0.001			3.2 (2.2, 4.5)	< 0.001
Sex						
Male	1.0 (reference)				1.0 (reference)	
Female	0.8 (0.7, 0.9)	0.003			0.4 (0.3, 0.6)	< 0.001
Residence						
Urban	1.0 (reference)		1.0 (reference)		1.0 (reference)	
Rural	2.0 (1.7, 2.5)	<0.001	1.7 (1.4, 2.0)	<0.001	1.4 (1.1, 1.8)	0.003
Education						
College/university or more	1.0 (reference)		1.0 (reference)		1.0 (reference)	
High school	2.1 (1.4, 3.2)	<0.001	2.2 (1.5, 3.3)	<0.001	2.0 (1.4, 3.1)	0.001
Middle school	3.8 (2.6, 5.6)	<0.001	3.3 (2.2, 4.9)	<0.001	2.9 (1.9, 4.5)	< 0.001
Elementary school or less	6.5 (4.6, 9.1)	<0.001	4.5 (3.1,6.6)	<0.001	4.0 (2.7, 6.0)	< 0.001
Income (quartile)						
4th	1.0 (reference)		1.0 (reference)		1.0 (reference)	
3rd	1.2 (0.9, 1.6)	0.25	1.1 (0.9, 1.5)	0.35	1.0 (0.8, 1.4)	0.88
2nd	1.6 (1.2, 2.2)	0.002	1.3 (0.9, 1.8)	0.06	1.1 (0.8, 1.5)	0.71
1st	2.5 (1.9, 3.2)	<0.001	1.3 (1.0, 1.8)	0.03	1.1 (0.8, 1.4)	0.63
Alcohol consumption						
No	1.0 (reference)		1.0 (reference)		1.0 (reference)	
Yes	0.8 (0.7, 1.0)	0.07	1.1 (0.9, 1.3)	0.40	1.1 (0.9, 1.3)	0.57
Smoking						
Non-smoker	1.0 (reference)		1.0 (reference)		1.0 (reference)	
Smoker	1.1 (0.9, 1.3)	0.31	0.7 (0.5, 1.0)	0.03	0.6 (0.5, 0.9)	0.003
Sunlight exposure (hr)						
<5	1.0 (reference)		1.0 (reference)		1.0 (reference)	
≥5	1.8 (1.5, 2.2)	<0.001	1.5 (1.2, 1.8)	<0.001	1.1 (0.9, 1.4)	0.29
Obesity (body mass index, kg/m^2^)						
< 25	1.0 (reference)		1.0 (reference)		1.0 (reference)	
≥25	1.1 (1.0, 1.3)	0.61	1.1 (0.9, 1.3)	0.30	1.1 (0.9, 1.3)	0.52
Diabetes mellitus						
No	1.0 (reference)		1.0 (reference)		1.0 (reference)	
Yes	1.2 (0.9, 1.6)	0.13	0.9 (0.7, 1.3)	0.25	0.9 (0.7, 1.2)	0.33
Hypertension						
No	1.0 (reference)		1.0 (reference)		1.0 (reference)	
Yes	1.7 (1.4, 2.0)	<0.001	1.1 (0.9, 1.3)	0.32	1.1 (0.9, 1.4)	0.38
Hypercholesterolemia						
No	1.0 (reference)		1.0 (reference)		1.0 (reference)	
Yes	0.9 (0.7, 1.2)	0.52	0.8 (0.7, 1.1)	0.19	0.9 (0.7, 1.2)	0.44

Values are presented as odds ratio (95% confidence interval).
